# Developing medical education through podcasts based on theories of personality

**DOI:** 10.1192/j.eurpsy.2023.2056

**Published:** 2023-07-19

**Authors:** B. Rooney

**Affiliations:** Medical student, Bristol University, Horsham, United Kingdom

## Abstract

**Introduction:**

More traditional lecture-based teaching has ruled medicine for generations. However, with the advancement of technology, a more modern approach to medical education can be developed. Podcasts are recordings, unlimited by time and space, that can act as extremely effective educational tools. Podcasts are a new approach that have the potential to transform the way medicine is taught.

**Objectives:**

This research project discusses the process of creating a podcast series for medical students based around the topic of personality theories. The aim of the project was not only to give medical students a more efficient and accessible way to learn, but also to tackle an undertaught topic in psychiatry. The podcast series I have developed explores the history of personality psychology, beginning with Greek philosopher Hippocrates and his theory of the 4 temperaments and travelling all the way up to the theories of the 20^th^ century, ending the series by discussing the most modern approach to assessing personality; trait theory. The topic of personality theories is essential psychology to grasp in order to fully comprehend psychiatric disorders learnt later in medical school.

**Methods:**

The process of creating educational podcasts did not come without its challenges. The vastness and complexity of information I came across was difficult and time consuming to narrow down and pick the most important points for medical students to understand. The biggest challenge I had was lack of clinical experience in psychiatry. Being involved in creating educational resources as a medical student, with little to no clinical experience in psychiatry, meant textbooks and primary literature were my key sources of information. However, I was aware this way of learning psychiatry was limiting; learning from textbooks alone may give a skewed picture of psychiatric conditions that can only be gained by seeing and learning from patients in the clinical environment. It allowed me to reflect on the use of online clinical videos in replacement of experiencing psychiatric placements.

**Results:**

The Podcasts themselves were crafted based on information from the most recent podcast research. They were made within the ideal timing and style to maintain audience engagement and allow listeners to process and retain new knowledge and make the most out of the learning style.

**Conclusions:**

They utilised Kolb’s learning style and allowed listeners to take an active role in the processing of new information by asking several questions throughout each episode. This technique especially gave learners opportunity to reflect on their own bias and change their perception about new concepts they were presented with. By the end of the production time, I realised the podcasts needed constant summaries in order to be successful learning materials.

**Disclosure of Interest:**

None Declared

